# Enhanced Bayesian modelling in BAPS software for learning genetic structures of populations

**DOI:** 10.1186/1471-2105-9-539

**Published:** 2008-12-16

**Authors:** Jukka Corander, Pekka Marttinen, Jukka Sirén, Jing Tang

**Affiliations:** 1 Department of Mathematics, Fänriksgatan 3B, Åbo Akademi University, Fin-20500 Åbo, Finland; 2 Department of Mathematics and Statistics, P.O. Box 68, Fin-00014 University of Helsinki, Finland

## Abstract

**Background:**

During the most recent decade many Bayesian statistical models and software for answering questions related to the genetic structure underlying population samples have appeared in the scientific literature. Most of these methods utilize molecular markers for the inferences, while some are also capable of handling DNA sequence data. In a number of earlier works, we have introduced an array of statistical methods for population genetic inference that are implemented in the software BAPS. However, the complexity of biological problems related to genetic structure analysis keeps increasing such that in many cases the current methods may provide either inappropriate or insufficient solutions.

**Results:**

We discuss the necessity of enhancing the statistical approaches to face the challenges posed by the ever-increasing amounts of molecular data generated by scientists over a wide range of research areas and introduce an array of new statistical tools implemented in the most recent version of BAPS. With these methods it is possible, e.g., to fit genetic mixture models using user-specified numbers of clusters and to estimate levels of admixture under a genetic linkage model. Also, alleles representing a different ancestry compared to the average observed genomic positions can be tracked for the sampled individuals, and *a priori *specified hypotheses about genetic population structure can be directly compared using Bayes' theorem. In general, we have improved further the computational characteristics of the algorithms behind the methods implemented in BAPS facilitating the analyses of large and complex datasets. In particular, analysis of a single dataset can now be spread over multiple computers using a script interface to the software.

**Conclusion:**

The Bayesian modelling methods introduced in this article represent an array of enhanced tools for learning the genetic structure of populations. Their implementations in the BAPS software are designed to meet the increasing need for analyzing large-scale population genetics data. The software is freely downloadable for Windows, Linux and Mac OS X systems at .

## Background

The past decade has provided an upsurge of methods and software enabling Bayesian statistical analyses of the ancestry and the current genetic structure of natural populations using a variety of molecular information sources, such as microsatellite and single-nucleotide polymorphism markers, as well as mitochondrial and house-keeping DNA sequences, to name a few. Reviews of the existing methods (see e.g. [1-3]) illustrate the jungle of software that can be in general exploited to infer ancestral patterns, migration and genetic isolation of subgroups of samples using Bayesian inference, e.g., BAPS [4-9], BAYES [10,11], BayesAss+ [12], GENECLUST, TESS [13], GENELAND [14-17], InStruct [18], NEWHYBRIDS [19], PARTITION [20], STRUCTURAMA [21], STRUCTURE [22,23]. Most of these methods rely on Markov chain Monte Carlo (MCMC)-computation in ways that have become more or less standard for modern Bayesian analysis [24].

We have earlier demonstrated the versatility of enhancing Bayesian computation through incorporation of analytical integration techniques into the stochastic search over the space of putative models, in the current applied context [4,5,8], in more general bioinformatics pattern recognition problems [25], as well as from a more theoretical statistical perspective [26,27]. It is apparent from the generic developments within molecular biology, that the statistical and computational methods used for the analyses of molecular datasets must evolve to meet the challenge stated by continuously increasing sizes of samples and the amount of molecular information characterized for them. With the cost of DNA sequencing decreasing rapidly and with the increasing number of molecular markers available for non-model organisms, a large number of research areas will face the need of feasibly applicable statistical tools to the myriad of questions related to the genetic population structure. It is evident both from the theoretical and applied literature concerned with Bayesian model learning and MCMC-computation, that the existing standard methods, such as the Gibbs sampler and reversible-jump Metropolis-Hastings algorithm [24], are not as such able to handle feasibly the challenge stated by large (e.g. at least one thousand samples) and complex (e.g. moderate to large number of mixture distribution components) molecular biological datasets [25,26,28]. By the latter we refer to datasets containing at least 10 and up to several hundreds of hidden groups or clusters.

In most of the subsections of METHODS, we introduce a number of new statistical tools that are implemented in the most recent version of the BAPS software, to further enhance the possibilities of exploring complex patterns associated with the evolution of populations from a genetics perspective. The novel features are then demonstrated through a set of analyses of molecular data sets in the RESULTS section.

## Methods

### Fitting genetic mixture and admixture models using a fixed number of populations

#### Motivation

Earlier introduced models and the associated stochastic estimation algorithms available in BAPS have been based on an approach where the number of genetically diverged subpopulations (i.e. genetic clusters) underlying the sample material have been learned statistically, given some reasonable *a priori *upper bounds determined by the user. While such an approach is relevant for most applications, there are situations in which it would be more appropriate to use fixed numbers of clusters specified by auxiliary information available. For instance, the sampling scheme might very likely be known to violate assumptions utilized for most genetic clustering methods, namely that the individuals represent fairly unrelated samples from the target population. With closely related samples it may happen that the estimate of the number of clusters is strongly biased, as the assumption of the Hardy-Weinberg equilibrium fails for such data. By fixing the cluster numbers within a certain confidence range, one can avoid spurious inferences about population structure due to genetic relatedness between samples. Also, biological auxiliary information may limit the number of possible ancestral sources of sampled individuals, say, into two alternatives, in which case it would be beneficial to restrict the analyses by not fitting models with three or more clusters. Such an approach is particularly useful when the molecular data are relatively sparse and the level of genetic differentiation between the ancestral sources is weak.

#### Description

In the stochastic partition representation utilized in [1,4,8], a genetic mixture model for a population consisting of *k *panmictic parts is determined by a partition *S *= (*s*_1_, ..., *s*_*k*_), which assigns *n *sampled individuals into *k *non-empty clusters. Using the molecular information available for the individuals, the inferences about the genetic mixture are obtained from the posterior distribution

(1) $$p\text{(}S\text{|}data\text{) }=\;p(data|S)p(S)/\sum_{S\in \Theta }p(data|S)p(S),$$

where Θ is the space of possible genetic structures (partitions), *p*(*data*|*S*) is the marginal likelihood of the molecular data for the genetic mixture *S*, and *p*(*S*) is the prior distribution of this structure parameter, which can be defined in various ways, depending on the availability of biologically relevant non-molecular information.

In the earlier introduced BAPS models, five different choices are available for specifying the *a priori *uncertainty about the genetic mixture in terms of *p*(*S*). Firstly, a default uninformative choice sets the prior equal to a uniform distribution over the set of genetic structures that are only restricted by an integer *K *(or a set of distinct integers *K*), 1 <*K *≤ *n*, an upper limit for the number of panmictic components thought to be feasible for the investigated population. The structure *S *which maximizes the posterior distribution (1) over this set is then sought using a stochastic search algorithm. Secondly, a more informative prior can be derived from the information provided by a commonly applied sampling design, where the strategy is to collect individuals from a number of geographically limited areas, yielding local sample populations. A uniform prior distribution can then be specified over the set of genetic structures satisfying the constraint that any subset of individuals with an origin in the same local sample population should not be assigned into separate clusters. The second prior thus corresponds to clustering of sample populations (or similar groups of individuals) instead of the individuals. Although such a prior for genetic structures is quite restrictive, it is still useful, in particular when the molecular information in the likelihood is very sparse (say 1–5 marker loci), or when the levels of genetic distances among populations are rather low.

The third and fourth prior alternatives are otherwise analogous to those two already presented, except that the prior distribution over different genetic structures is not uniform, but favors spatial smoothness in the clustering solution. Such priors were derived in [9], who introduced a spatial clustering model for molecular marker data. Under these alternatives it is assumed that sample coordinates are available either for the collected individuals, or for the local sample populations, depending on the level at which the clustering is to be performed. The spatial prior distributions are then specified under the constraint that the upper bound *K *is determined application-wise, and the corresponding posterior optimal number of clusters within the range [1, *K*] is estimated using the stochastic learning algorithm. The particular advantage of the spatial priors in contrast to the uniform distribution is that they provide more weight for clustering solutions that are expected to be sensible from the biological perspective *a priori*, which strengthens the inferences for sparse molecular data. However, for strongly informative markers and clear genetic boundaries in the sample, the two approaches will provide highly similar inferences, as the prior distribution will be dominated by the marginal likelihood of the molecular data.

The final alternative to specifying the prior distribution over the genetic structures is provided by considering trained clustering, where it is assumed that separate baseline data consisting of individuals with known origins is available for at least some of the possible genetic origins underlying the sample data. The molecular information from the baseline is then utilized to update information concerning the allele frequencies in the corresponding subpopulations, which is further used to assess models that assign sample data to these and other putative origins in various configurations. The number of clusters present in the sample data is here otherwise learned similarly as for the previously described models, i.e. under an *a priori *determined upper bound *K*.

To provide more flexibility for the genetic mixture estimation, we have in BAPS 5.1 extended each of the above modelling approaches to allow for a fixed number of clusters to be used instead of letting the algorithm learn the value under a given upper bound *K*. This means that the prior distribution is otherwise analogously determined as according to the five described alternatives, except that it gives non-zero probabilities only for the genetic structures that have exactly the specified number of clusters. It is worth emphasizing that the stochastic maximization of the posterior is still done in a similar fashion as for the models with an upper bound *K*, by using the analytically calculated marginal likelihoods to evaluate the clustering solutions, however, under the constraint that the number of clusters does not change during the iterations of the estimation algorithm. The particular advantage of utilizing the analytical approach is that it avoids the Monte Carlo error for the estimation of a marginal likelihood for a Bayesian model. Such errors can be substantial even for a relatively large number of Monte Carlo iterations, when the number of clusters is at least considerable and/or the number of samples assigned in a cluster is small, due to the increased instability of the allele frequency samples from the corresponding posterior distribution. In situations where it is still of interest (and appropriate) to compare the statistical adequacy of estimated genetic structures having different numbers of clusters, the obtained maximal marginal likelihood values *p*(*data*|*S*) for the different solution can be used as previously.

### Comparing a *priori *specified biological hypotheses about the population structure

When molecular methods are applied in population genetics, it is common that previously gained context-specific knowledge, or even more general theories, may suggest a range of alternative genetic structures that are *a priori *considered plausible for some set of sample data. Under such circumstances, fitting of genetic clustering models as presented previously can be an inconvenient and indirect way of comparing the existing *a priori *hypotheses about the genetic structure. To facilitate such comparisons, we have implemented a possibility to do the analysis using the posterior probabilities for the hypotheses corresponding to the fixed genetic mixture models. Let *H*_*i*_, *i *= 1, ..., *m*, be *m *hypotheses, each specifying unambiguously a value for the partition *S *in a genetic mixture model, and *p*(*H*_*i*_) the corresponding prior probabilities for the hypotheses. The posterior probabilities for the hypotheses are then defined as

(2) $$p\text{(}H_{i}\text{|}data\text{)}=p(data|H_{i})p(H_{i})/\sum_{i=1}^{m}p(data|H_{i})p(H_{i}),$$

and provide a direct way of comparing the relative plausibility of the competing structures.

### Discovering alleles with a deviating ancestry

#### Motivation

In our earlier work [4], we introduced a two-stage strategy to inferring admixture events for individuals, where a genetic mixture model is first fitted to the data, and the likelihood of admixture for each individual is then considered conditional on the number of putative ancestral sources in the mixture. This strategy was proposed as a way of handling the weak identifiability resulting from admixture models where the number of ancestral sources is inferred simultaneously with the admixture coefficients of the individuals, as this may lead to spurious inferences, see [4].

While admixture models are useful for shaping our understanding about the past events in a population, they have a somewhat narrow scope in certain contexts, where it is of interest to identify at which loci an individual might have alleles representing a deviating ancestry. Namely, as the admixture framework is focused on the average proportions of genomic content that can be assigned to particular ancestral sources, it does not directly reveal which alleles or nucleotides should be considered to carry statistically conclusive evidence for an ancestry distinct from the origin assigned to an individual in a genetic mixture analysis. Such an inference task is particularly relevant in analyzing bacterial population structures, since horizontal gene transfer between groups of different origins play an important role in shaping the bacterial gene sequences. Also, for suspected hybrid individuals, it may be of interest to explore how many and which genomic regions show signs of recombination if data are available from the parental lineages. Moreover, migration events in a relatively distant past, for which the traces present in the molecular data are already quite diluted, may go undetected in an admixture analysis when the focus is on average proportions of genomic content, especially if a large number of loci is considered.

**Figure 1:**
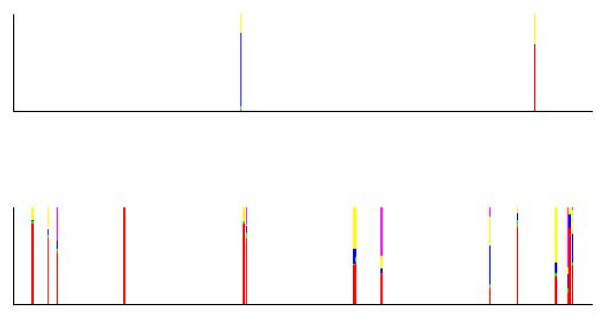
**Posterior probabilities of the origins of alleles for an admixed individual from the population labelled C/D, who was assigned into the cluster with green label in the genetic mixture analysis.** The posterior probabilities are only shown for the alleles where the log_e _Bayes factor for an ancestry deviating from the origin labelled green exceeds the default threshold (2.30). For simplicity of the visualization, the genotype data are assumed ordered, such that the lower and upper panels correspond to chromosome 1 and 2, respectively.

**Figure 2:**
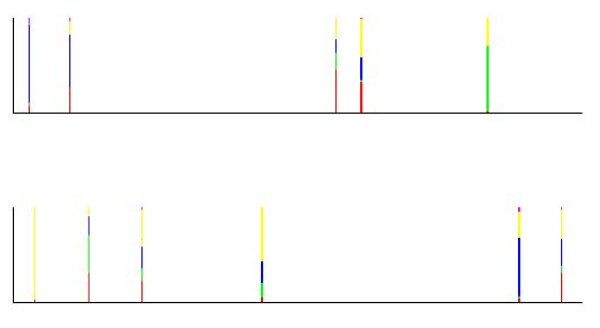
**Posterior probabilities of the origins of alleles for an individual with pure ancestry in the population labelled E, who was assigned into the cluster with magenta label in the genetic mixture analysis.** The posterior probabilities are only shown for the alleles where the log_e _Bayes factor for an ancestry deviating from the origin labelled green exceeds the default threshold (2.30). For simplicity of the visualization, the genotype data are assumed ordered, such that the lower and upper panels correspond to chromosome 1 and 2, respectively.

In order to complement the picture painted by an admixture analysis about the past events in a population, we introduce here a simple statistical tool which can be exploited to discover alleles with a deviating ancestry, given the results for an earlier estimated genetic mixture model. Our approach is based on the use of Bayes factors combined with predictive likelihoods to compare the evidence for alternative ancestral sources at each marker locus observed for a particular individual (examples are provided in Figures 1 and 2). In the implementation of this tool it is possible for a user to determine the level of conclusive evidence for deviating ancestry, while the default threshold is chosen according to the categories advocated in the theoretical literature [26]. We note that as the tool treats the data from all loci separately, it serves primarily as an exploratory method. In particular, for studies of bacterial populations based on DNA sequences from multiple genes, it is possible to perform more detailed analyses, for instance, using the model introduced by [29].

#### Description

To consider the statistical uncertainty related to the ancestry of an observed allele (or a nucleotide) at a particular locus (site), we utilize in a combined manner Bayes factors [30] and posterior probabilities for the allele frequencies in the putative origins to efficiently explore the molecular data, such that loci carrying plausible evidence for deviating ancestry are discovered.

We assume that a genetic mixture analysis is performed prior to an attempt to discover alleles with a deviating ancestry, to identify the possible putative origins of the alleles. Such an analysis may be done either using the approach where the suitable number of clusters is learned by BAPS, or by specifying the number of clusters in advance. The number of distinct ancestral origins used here is denoted by *k*. Let *a*_*ij *_be an observed allele for an individual *i *at the locus *j*, and further, let *c *denote the cluster into which this individual was assigned in the genetic mixture analysis. For notational simplicity, we assume here that the data are haploid. Diploid data is dealt with accordingly by considering both the alleles at a single locus separately. The strength of evidence for an ancestry distinct from *c *is assessed by the maximal Bayes factor

(3) $$\text{BF}=\underset{c'\neq c}{\arg\max}\frac{p\text{(}a_{ij}\text{|}s_{c'})}{p\text{(}a_{ij}\text{|}s_{c})},$$

where *p*(*a*_*ij*_|*S*_*c*_) is the predictive likelihood of allele *a*_*ij *_in the genetic origin represented by cluster *c *after removing *a*_*ij *_from the molecular data obtained from *s*_*c *_at locus *j*, and similarly, *p*(*a*_*ij*_|*S*_*c*'_) is the corresponding predictive likelihood for cluster *c*' (where no removal is necessary as individual *i *is not included in *s*_*c*'_). The predictive likelihood is the probability of observing the allele *a*_*ij *_within a particular genetic origin, when the remaining uncertainty about the allele frequencies at locus *j after *the molecular data obtained from individuals assigned in the particular cluster has been taken into account. Thus, these predictive likelihoods reflect in a statistically sensible manner both the possibly varying levels of polymorphism and missing data over the loci. To identify loci where the statistical evidence for a deviating ancestry is at least substantial, we use 2.3 as a default threshold for the log BF, as suggested by the guidelines discussed in [30]. This threshold implies that the particular allele under consideration is at least 10 more likely to be observed in a cluster other than *c*. However, in our software implementation it is possible to choose the threshold freely, for instance, if only loci with very strong evidence of a deviating ancestry are sought. Under the reference Dirichlet prior utilized for the allele frequencies in the earlier BAPS works, the above predictive likelihoods can be written as

(4) $$p\text{(}a_{ij}\text{|}s_{c'})=\frac{\Gamma (\sum_{l=1}^{N_{A(j)}}\alpha_{j}+n_{c'jl})}{\Gamma (\sum_{l=1}^{N_{A(j)}}(\alpha_{j}+n_{c'jl})+1)}\prod_{l=1}^{N_{A(j)}}\frac{\Gamma (\alpha_{j}+n_{c'jl}+I(a_{ij}=l))}{\Gamma (\alpha_{j}+n_{c'jl})},$$

where Γ(·) is the gamma function, *n*_*c*' *jl *_is the number of copies of allele *l *(*l *= 1, ..., *N*_*A*(*j*)_) at locus *j *observed in the cluster *c*', *I*(*a*_*ij *_= *l*) is an indicator function of *a*_*ij *_being equal to *l*, and α_*j *_is a Dirichlet prior hyperparameter equal to 1/*N*_*A*(*j*)_.

To aid the summarization and interpretability of the allele screening results for any individual of interest, we have implemented the Bayes factor-based discovery tool in such way that it displays a visual map of the loci where the threshold of (3) is reached. The statistical basis of this map is similar to the admixture plots, where an individual is shown as a multicolored vertical bar, such that the relative height of each color is equal to the estimated percentage of the genome having an ancestry from the corresponding origin. Here, similar bars are drawn for each locus (on two separate images for diploid data) where the maximal Bayes factor value is larger than the threshold; however, with the distinction that all the bars represent the inferences for the same individual. The relative height of the color corresponding to ancestral origin labeled by *c*' is equal to the posterior probability under the uniform prior over the *k *possible origins, which is calculated analytically as

(5) $$\frac{p\text{(}a_{ij}\text{|}s_{c'})}{\sum_{\underset{c'\neq c}{c'=1}}^{k}p\text{(}a_{ij}\text{|}s_{c'})+p\text{(}a_{ij}\text{|}s_{c})}.$$

For examples, see Figures 1 and 2, which are introduced in the RESULTS section. If a conservative approach to interpreting the above values is taken, then only cases where the bar area is solely or almost solely represented by a single color, are considered as evidence for a deviating ancestry of a particular allele. However, such a strict limitation would tend to ignore alleles that are brought into a population by migration or admixture (even several generations earlier), but which are common in more than one alternative ancestral origin.

It should be noticed that making inferences about the origin of an allele (or genotype) at any particular single locus is *per se *more challenging than the admixture analysis, which combines the information provided over all considered loci. This is due to the fact that the allele frequencies at a single locus in the different possible populations of origin have to be quite distinct to yield conclusive evidence for a deviating ancestry. Moreover, especially for highly polymorphic loci, only a fraction of the individuals representing a specific ancestral origin tends to carry alleles that are markedly characteristic for that origin, at a given locus. Thus, it is not realistic to expect that statistically conclusive evidence for deviating ancestry can be obtained for a large number of loci, unless the populations in question are strongly differentiated in genetic sense.

### Admixture analysis under a genetic linkage model

#### Motivation

As discussed in the previous section, [4] developed a Bayesian approach to the estimation of the levels of admixture in individuals' genomes from sample data with unlinked molecular markers. Here we describe an extension of this approach to linked molecular data under the linkage model introduced by [5]. Also, we improve the simulation-based estimation framework of [4] to both reduce the computational burden for large datasets and to obtain more accurate *p*-value estimates in situations where the amount of missing molecular data varies considerably among the observed individuals. These computational enhancements are applicable to the admixture estimation for unlinked marker data as well.

Assume that the molecular data cover *m *genomic regions, e.g. genes, such that the regions are considered unlinked and that the dependencies among the sites within each individual region are represented by a Markov structure as in [5]. Then, it is possible to find an analytical expression for the joint probability of an observed genetic profile over the considered regions and to use Monte Carlo sampling for the allele frequencies conditional on an estimated genetic mixture model. Consequently, principally the same strategy to finding marginal maximum *a posteriori *estimates of the admixture coefficients and assessing their significance, as in [4], can be used.

#### Description

Let *ω*_*i *_= (*ω*_*i*1_, ..., *ω*_*ik*_), $\sum_{c=1}^{k}\omega_{ic}=1$ be a vector of admixture coefficients representing the proportions of the genome of individual *i *with ancestry in the corresponding origins. Assume that the observed molecular data are represented by either *n*_*g *_genomic regions (DNA sequence data) or linkage groups (linked marker data). The linkage model introduced in [5] represents the dependencies by Markov structures, under which the joint probability of the data over the considered sites or loci can be expressed using an analytical factorization. Let *p*_*c*_(**x**_*ig*_|*ω*_*i*_) be such a joint probability of the data from the *g*th region for individual *i *within the subpopulation *c*. Then, the admixture model likelihood for all the data **x**_*i *_from *i *is determined by

(6) $$p(x_{i}|\omega_{i})=\overset{n_{g}}{\underset{g=1}{\prod }}p(x_{ig}|\omega_{i}),$$

where

(7) $$\begin{array}{lll} p(x_{ig}|\omega_{i}) & = & \frac{{}_{\prod q\in Q_{g}}p(x_{iq}|\omega_{i})}{{}_{\prod s\in S_{g}}p(x_{is}|\omega_{i})}\\ & = & \frac{{}_{\prod q\in Q_{g}}\sum_{c=1}^{k}\omega_{ic}p_{cq}}{{}_{\prod s\in S_{g}}\sum_{c=1}^{k}\omega_{ic}p_{cs}}, \end{array}$$

where **Q**_*g *_and **S**_*g *_are sets of all cliques and separators in region *g p*_*cq *_is the probability of clique *q *in cluster *c*, and *p*_*cs *_is the probability of separator *s *in cluster *c*

Here we use the same prior for *ω*_*i *_as in [4], and obtain the marginal posterior mode estimates by numerical maximization combined with a Monte Carlo simulation, to account for the uncertainty about the probabilities *p*(**x**_*i *_| *ω*_*i*_) given a genetic mixture estimate. It follows from the basic properties of the Dirichlet-distribution and decomposable graphical factorizations (e.g. [31]) that realizations from the posterior of *p*(**x**_*i *_| *ω*_*i*_) can be simulated using an ordinary Monte Carlo approach even under the linkage model.

After the estimation of the admixture coefficients for the individuals, a simulation procedure is carried out in order to assess their statistical significance. By simulating reference individuals without admixture from the populations identified in the mixture clustering, we can estimate the distribution of admixture coefficients under the no-admixture hypothesis. In particular, we can assess the probability that the non-zero admixture coefficients obtained for the individuals in the data could have arisen by chance alone, and not represent a real admixed background.

To reduce notably the simulation burden for large and complex datasets, we use a simple pre-filtering to remove those individuals for which there is definitely not enough statistical evidence of admixture to obtain significance at 5%-level. The filtering is accomplished by investigating the difference in the maximized log-likelihood between the admixture hypothesis and the no-admixture hypothesis. If this difference is less than 3, it is concluded that there is not enough evidence in the likelihood to obtain significance for admixture, and the individual is not considered further by simulation. The simulation-based *p*-value (see below) for such individuals will be set to unity. Notice that the chosen filtering threshold represents a very liberal significance limit in an asymptotic likelihood ratio test with the minimum possible degrees of freedom, and thus, no cases where there are non-negligible chances of obtaining a *p*-value smaller than 5% will be left out of the consideration.

When non-admixed reference individuals are simulated from a subpopulation identified in the genetic mixture estimation, the fact that the levels of missing data may vary between individuals should be explicitly taken into account, which was not done by [4]. Otherwise, elevated levels of significance may be obtained under some circumstances, where the degree of missing information in the genetic profiles of certain individuals allows spuriously high posterior mode admixture coefficients to arise for a false origin. To prevent such events, we have developed a strategy where each estimated subpopulation is further divided into clusters based on the proportions of the missing alleles of the individuals assigned to that subpopulation. In this clustering the individuals are allowed to be grouped into maximally three subsets using the proportions of missing alleles as evidence in the modelling approach derived in [32]. Notice, however, that the subgroups are not forced by the method, i.e. a single cluster may result from this procedure, if the observed individuals are relatively homogeneous with respect to the levels of missing data.

Given the obtained subgroups with respect to varying degrees of missing information in the genetic profiles of individuals assigned to a specific cluster in the genetic mixture analysis, a number of non-admixed reference individuals are simulated for each subgroup. However, this simulation effort is used only for those clusters, where the pre-filtering described above has not removed all individuals, as there is otherwise no need to obtain significance limits for the admixture estimates. The admixture coefficients are estimated for the simulated individuals in exactly the same manner as for the individuals in the observed data. To summarize these analyses, a simulation-based *p*-value is given for each individual in the data, and it corresponds to the proportion of the simulated individuals from the same population and with the same level of missing data, whose estimated admixture coefficient *ω*_*ic *_for their true population is smaller than or equal to the estimated coefficient for the investigated individual. Thus, this *p*-value reflects how small a proportion of the genome can be assigned to the true ancestral origin of an individual by chance, while taking into account the uncertainty about the allele frequencies in the underlying population and the extent of information in the observed genetic profile.

### Enhancements in the computational architecture and presentation of results

As model-based clustering and admixture analysis are statistically and computationally very challenging problems, in particular for large and complex datasets, we have improved the computational architecture inside BAPS to facilitate fitting of the Bayesian models. Firstly, as discussed in the previous section, the pre-filtering approach developed for the admixture analysis reduces considerably the number of simulation steps when there exists at least a moderate number of individuals in the data with negligible evidence of admixture. Secondly, we have optimized the information processing in the clustering algorithms, such that the computation time required for fitting a linkage clustering model to a large dataset has decreased by approximately 80% compared to the earlier version (4.14) of the software. Finally, we have introduced the possibility to use script files to run analyses, such that the replicate runs of the genetic mixture clustering or the admixture reference simulations can be performed simultaneously in separate computers. The analytical approaches utilized in our models enable a subsequent aggregation and comparison of the results from these estimation processes, using built-in features of the software interface. To facilitate the extraction of information from the genetic mixture analyses, both graphical and numerical summaries of the relationships among inferred clusters can be obtained. We have in particular implemented the possibility to draw UPGMA and Neighbor-Joining trees (see, e.g. [33]) using several different visual types (square, angular, radial, phylogram) and distance measures (Kullback-Leibler divergence, Nei's and Hamming distance).

## Results

### Fitting genetic mixture and admixture models using a fixed number of populations

To illustrate the flexibility provided by the approach based on a fixed number of clusters that are fitted to the molecular data, we consider a dataset from [34]. The data consists of 90 individuals collected from three distinct population isolates that all had the same origin in a specific founder population 20 generations earlier. The sample size from each population isolate is 30, and these individuals represent 10 sibships with three siblings in each. The observed molecular data comprise 15 unlinked microsatellite loci for which alleles were simulated by [34] using a realistic setup of founder allele frequencies with a sensible mating scenario. The estimated maximum posterior genetic structure based on the individual level clustering model for unlinked markers with exactly three clusters is nearly equal to the underlying grouping, showing only a single incorrectly assigned individual. Similarly, to investigate how well the sibling trios could be identified from the data, the posterior optimal structure with 30 clusters was estimated, and also this estimate misplaced a single individual.

To pinpoint the advantages gained from the analytical integration approach combined with the stochastic search, we compared these results to those reported in [34] based on STRUCTURE analyses of the same data. Notably, at the population isolate level (*K *= 3) the results are quite similar, *i.e*. STRUCTURE assigned incorrectly only three individuals. However, at the sibship level (*K *= 30) the genetic boundaries identified by STRUCTURE are considerably blurred for approximately half of the trios (Figure 5 in Estimating genealogies from unlinked marker data: A Bayesian approach [34]). This difference arises likely from the Monte Carlo error related to the estimation of underlying allele frequencies, as the sample sizes in the trios are small. We also performed admixture estimation with BAPS for these data, both conditional on the estimated genetic mixture with *K *= 3 and *K *= 30. Neither of the two analyses yielded any significant admixture estimates at 5% significance level, i.e. no false positive admixture cases were detected for these data.

### Comparing *a priori *specified biological hypotheses about the population structure

To illustrate the introduced feature for a direct comparison of biological hypotheses concerning genetic population structure, we consider microbiological data from [35]. The data consist of 18 seawater microbial community samples from Tokyo Bay area fingerprinted using terminal restriction fragment length polymorphisms (T-RFLP). The microbial composition of a sample was characterized by a number of markers, each of which represents the quantitative abundance of a certain bacterial species. There are 71 marker loci in total and we consider them here in the same binarized form as referred to in [35]. The 10 sample sites are located in the Keihin canal approximately linearly from a position containing an open sewage pipe end towards the canal's outlet to Tokyo Bay, and, at 8 out of the 10 sites, both surface and bottom were successfully extracted.

The environmental factors present in the sampling design suggest that the prominent salinity gradient between surface and bottom water might lead to a genetic separation of the microbial communities existing at the two water levels. On the other hand, the form of the canal and the waste water load nearby the sewage pipe might create a separation of the area closest to the sewage pipe (two samples) from the narrow canal area (10 samples), which would further be separated from the remaining samples, as these are located in the open bay area and correspondingly affected more by the sea currents. Thus, these environmental factors may suggest a genetic structure with three clusters in the data. Finally, given the relatively high levels of nutrient loading in the water throughout the canal and bay area, it could be plausible that the genetic structure of the sampled region consists solely of a single cluster. To compare these hypotheses, we assigned each an equal *a priori *probability (*p*(*H*_*i*_) = 1/3), and calculated the posterior probabilities using the model of unlinked markers at the individual level. The resulting probabilities for the three hypotheses are approximately 1.00, 0.00 and 0.00, respectively, which indicates that for these data the salinity differences seem to have a greater role in shaping the microbial communities, than the closeness to the waste water source.

### Discovering alleles with a deviating ancestry

In our earlier work [4] we created a simulated admixture scenario based on extensive human microsatellite data from [36], where first- and second-generation admixture cases were combined with individuals representing non-admixed ancestry. The allele frequencies for the underlying simulated populations were set equal to the posterior mode estimates for the clusters inferred by BAPS analysis of the original data. Here we use an analogous simulation framework to illustrate the possibilities for the discovery of alleles with a deviating ancestry. Our simulated data consists of 700 individuals in total, for which genotypes were generated over 377 microsatellite loci using the allele frequencies in the earlier inferred BAPS clusters (Eurasia, Africa, Oceania, East Asia and America). In addition to the individuals with pure ancestry in one of the underlying five populations, the dataset contains three groups of individuals whose parents represent distinct genetic origins, as well as one group for which the parents themselves have admixed ancestry.

**Figure 3:**
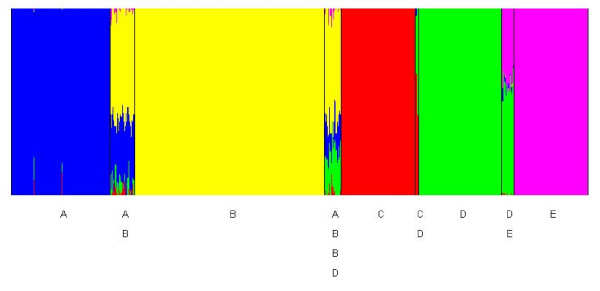
**Posterior estimates of the admixture coefficients for 700 individuals with 377 microsatellite loci simulated using five underlying populations indicated by the black vertical lines (A = Eurasia, B = Africa, C = Oceania, D = East Asia, E = America).** The populations with two labels indicate that the individuals are admixed between the two origins (one parent from each population). The populations with four labels indicate that the individuals have ancestry in the corresponding populations (admixed parents). The allele frequencies used in the simulation are the posterior mode estimates under a Dirichlet prior from the human data reported in [32] using the same clusters as in [4].

The simulated human microsatellite data were first analyzed using a genetic mixture model for unlinked markers with the *a priori *upper bound for the number of clusters set equal to 10. The resulting posterior mode clustering was then used for admixture inference and the results are shown in Figure 3. In this graphical presentation we have used the default option in BAPS, where the estimated admixture coefficients are distinct from pure ancestry only for individuals assigned with a significant *p*-value (the default threshold value being 5%). This type of a presentation facilitates the visual interpretation of the results, as statistically spurious admixture coefficient estimates distinct from zero are filtered out. It is seen that all individuals with an admixed ancestry were considered significant, and that additionally two out of 430 individuals with a pure ancestry (population A, blue color) were spuriously inferred to be admixed.

In Figure 1, putative loci associated with a deviating ancestry are shown for an individual with admixed ancestry between the populations marked with the red (population C) and green (population D) colors in Figure 3. In the genetic mixture analysis this individual was assigned to the green cluster, and thus, the Bayes factor assesses the statistical evidence for an ancestry distinct from the population D for each allele at each locus. In Figure 1 the alleles at those loci out of the 377 in total are shown, where the log_e _Bayes factor exceeds the default threshold (2.3), such that the relative height of each color in the bars corresponds to the posterior probability of the allele having its origin in the population with the corresponding color. For the convenience of visualization and numerical summaries of the Bayes factors, it is assumed that diploid molecular data are ordered and that the lower and upper panels of the figure correspond to the first and second chromosome, respectively. For datasets where this information is not available, the graphical representation simply reflects the ordering of the alleles within the genotypes in the dataset.

The role of the posterior probabilities is here to aid the interpretation of the allele screening results based on the Bayes factors. Namely, they enable a simultaneous visualization of the pattern of the deviating ancestry suggested for a particular individual, and also, they can be used to further assess the statistical evidence. As a conservative strategy, it is suggested that the evidence for a deviating ancestry is considered strong, if the Bayes factor exceeds at least the default threshold *and *the posterior probability of any of the suggested deviating origins of the allele is at least 80%. Albeit this strategy reduces the statistical power to detect interesting alleles with putatively deviating ancestry, it also reduces considerably the extent of false positives among the findings. From Figure 1 it can be concluded that fairly conclusive evidence for the ancestry in the population C (red) can be obtained for roughly 10 alleles scattered over the first chromosome that had its ancestry in population C (the lower plot). The log_e _Bayes factor values for the origin in the true population (C) vary approximately over the interval [2.60, 5.98] for these cases. It is also seen that false conclusive evidence for a deviating origin for any other ancestral source population is only obtained for a single allele (log_e _Bayes factor 3.01), which is inferred to have ancestry in the population labelled A. The pattern in Figure 1 can be compared with that arising for an individual with a pure ancestry in a population E, which is shown in Figure 2. Here, only two alleles are suggested to have fairly conclusive evidence for a deviating ancestry by the combination of the Bayes factors and posterior probabilities, and in contrast to the previous individual, no single alternative ancestral source is inferred to have contributed significantly over several locations in the genome. However, it may still be of biological interest to screen for putatively deviating loci using this tool, even under a similar situation where no significant signs of admixture have been detected for a particular individual.

### Admixture analysis under a genetic linkage model

To reasonably mimic characteristics of datasets for which the linkage mixture and admixture models are intended, we use a real bacterial data published in [37], to generate a set of synthetic multilocus DNA sequences with which the admixture inference is performed. The real data consists of DNA sequences for seven housekeeping genes for a sample of 120 strains representing the *Burkholderia cepacia *complex, with the total length of 2773 bases over all the genes. Our simulated dataset was generated by imitating the level of observed molecular divergence among these *Burkholderia *strains. Firstly, an arbitrary strain was selected as a global strain profile, being the ancestor of the whole simulated population. Then, local ancestors for three subpopulations were generated from the chosen global ancestor, by inserting mutations in the DNA sequence of the global strain, such that they occur at a rate of 0.035 per site. Thus, on average, the local ancestors are separated from the global strain by 0.035*2773 = 97 mutations. Finally, to obtain the synthetic data for the non-admixed strains, mutations were inserted into the DNA sequences of the local ancestors, such that they occur at a rate of 0.0054 per site, i.e. the strains are on the average separated from their local ancestor by 0.0054*2773 = 15 mutations. The numbers of generated strains with non-admixed ancestry were 25, 20 and 20, for the three subpopulations, respectively. Additionally, five strains with a considerable degree of recombination between the subpopulations 1 and 2 were included in the data. The molecular profiles of these strains were created independently of each other, such that at approximately 25% of the sites the observed bases were generated according to the mutation model from the local ancestor of population 2, and correspondingly, at approximately 75% of the sites from the local ancestor of population 1.

The simulated data with 70 strains described above were analyzed using the second-order Markov linkage clustering model of [5], with the *a priori *upper bound *K *for the number of clusters set equal to 10, and using five replicate runs of the stochastic estimation algorithm. The three underlying clusters were correctly discovered in the analysis, and we performed the linkage admixture analysis conditional on the obtained posterior optimal genetic mixture, where the admixed strains were allocated to the cluster corresponding to the first underlying population. In the admixture analysis based on the default number of simulations used in BAPS 5.1, all the admixed strains were assigned with *p*-values equal to 0.00, and all other strains with *p*-values equal to 1.00. This finding reflects the information content of the molecular data, as no false positive or negative results were obtained. The average estimated admixture coefficient for the ancestry in population 2 is 0.09 for the admixed strains, which underestimates to some extent the contribution of this origin to the genomes of the strains. However, it should be noticed that in this type of a DNA sequence data, many of the sites with a deviating ancestry in fact carry identical bases in all underlying populations, which means that they are not informative in the admixture likelihood, and thus, it is expected that the contribution from a deviating ancestral origin cannot be very precisely estimated, even under ideal circumstances from the statistical perspective.

### Illustrative example of challenging genetic mixture analyses

As a final illustration of the issues related to the information provided by the sampling design in genetic mixture clustering, we consider the recent human dataset from [38]. The data contains genotypes at 678 autosomal microsatellite loci for 1484 individuals collected from 78 geographically defined populations, out of which 24 represent native populations in Americas, and the remaining ones are localized in Africa, Eurasia and Oceania. First, we performed a genetic mixture analysis by clustering these data at the sample population level using the *a priori *upper limit *K *= 30 and 10 replicate runs of the estimation algorithm, which yielded the posterior optimal clustering solution with 11 clusters corresponding to random mating units (Table 1). [38] used the STRUCTURE software in various genetic admixture analyses of this data; however, they did not report results from any global analysis having more than six clusters, although their separate analysis of the Americas region revealed considerable substructure in that region, which is quite similar to the results of our genetic mixture analysis in Table 1. A similar strategy was earlier followed by [36], who reported for a subset of the data in [38] only global clustering solutions up to six clusters, due to convergence problems in the STRUCTURE estimation.

**Table 1:** Posterior mode clustering of the human data from [34] using the genetic mixture analysis at the sample population level in BAPS.

Cluster:	Included sample populations:
Cluster 1	Han, Han-NChina, Dai, Daur, Hezhen, Lahu, Miao, Oroqen, She, Tujia, Tu, Xibo, Yi, Mongola, Naxi, Cambodian, Japanese, TundraNentsi, Yakut
Cluster 2	Melanesian, Papuan
Cluster 3	Orcadian, Adygei, Russian, Basque, French, Italian, Sardinian, Tuscan, Mozabite, Bedouin, Druze, Palestinian, Balochi, Brahui, Burusho, Hazara, Kalash, Makrani, Pathan, Sindhi, Uygur
Cluster 4	Kogi, Arhuaco
Cluster 5	TicunaArara, TicunaTarapaca
Cluster 6	BantuSouthAfrica, BantuKenya, Mandenka, Yoruba, BiakaPygmy, MbutiPygmy, San
Cluster 7	Karitiana
Cluster 8	Piapoco, Maya, Chipewyan, Cree, Ojibwa, Kaqchikel, Mixtec, Mixe, Zapotec, Guaymi, Cabecar, Aymara, Huilliche, Guarani, Kaingang, Quechua, Zenu, Inga, Wayuu, Embera, Waunana
Cluster 9	Pima
Cluster 10	Surui
Cluster 11	Ache

**Figure 4:**
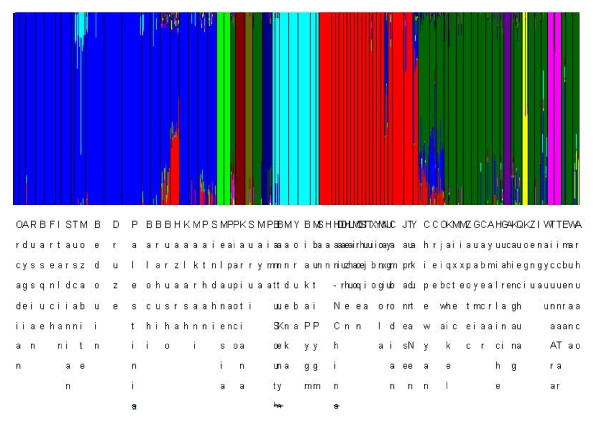
**Posterior admixture estimates for the human data reported in **[34]** based on the optimal genetic mixture estimate with 11 clusters under the BAPS uniform prior clustering model for individuals.**

To investigate the genetic structure of the human data at a finer scale, we also performed genetic mixture clustering at the level of individuals in BAPS, using a range of *a priori *upper bound values *K*. The challenge posed by a large-scale dataset with a complex structure, i.e. several small clusters associated with relatively low levels of genetic differentiation from the remaining populations, was clearly illustrated by these data. For small to moderate *a priori *upper bound values, the stochastic estimation algorithm used in BAPS was unable to detect more than six clusters in the individual level analysis, and the analytical comparison of the solutions' log marginal likelihood values revealed that they were inferior to the earlier derived estimate with 11 clusters (Table 1). To gain better understanding of the behavior of the stochastic estimation algorithm, we increased the *a priori *upper bound value successively, until the posterior mode estimates started to converge to the vicinity of the 11-cluster solution. From *K *= 500 upwards, new clusters were successively identified, and with *K *= 800, the algorithm was finally able to discover a genetic mixture with a higher posterior probability than the 11-cluster solution obtained in the clustering at the sample population level. This new optimum contained 11 clusters as well; however, some of the individuals were re-allocated into alternative clusters different from those containing the majority of their sample population data. We finally performed an admixture analysis conditional on the optimal genetic mixture estimate from the individual level analysis. The posterior mode estimates of the admixture coefficients significant at the 5% level using the default options in BAPS are shown in Figure 4.

## Discussion and conclusion

The apparent popularity of the Bayesian approach to solving inference problems in population genetics has led to a surge of interest to develop new models and methods for analyzing a variety of questions related to the genetic structure of a population, its intrinsic ancestral patterns and the assignment of individuals to distinct origins. Our experiences from the development of the approaches implemented in the BAPS software have crystallized the need for models and algorithms that reasonably scale up to large and complex datasets, both with respect to the time-complexity and the accuracy of the inferences.

Our example concerned with the extensive human dataset from [38] illustrated how difficult it can be to detect minor underlying clusters when the size of the clustering space increases considerably, unless these clusters are strongly separated from the remaining data in genetic terms. Even with the intelligent stochastic search operators implemented in BAPS, clusters may go undetected under such circumstances, unless the initialization phase manages to discover suitable existing boundaries within the user-specified limits (maximum number of allowed clusters).

It is important to notice that the information contained in the sampling design provides often a statistically powerful means for guiding the model estimation towards optimal clustering solutions, as illustrated by the comparison of the sample and individual level genetic mixture analysis of the human data. In fact, we also performed a modified genetic mixture clustering at the individual level, by starting the analysis from the optimum solution obtained from the sample population clustering. This analysis converged to the same global optimum with 11 clusters under the *a priori *bound *K *= 30, as the default BAPS analysis initialized using the prior boundary *K *= 800. This finding suggests that our estimation algorithms could be further improved by utilizing various strategies in the initialization phase, although the currently implemented methods have been successfully applied for a wide range of challenging applications.

It is clear that any of the existing Bayesian approaches to genetic population structure inference needs to evolve to meet the challenge from the large-scale and/or genome-wide genotyping that is available for a continuously increasing number of organisms. Here we have discussed an array of statistical tools that represent a step towards that direction; however, a multitude of innovative strategies for model learning, such as Bayesian variational estimation and more intelligent ways of screening for initial model estimates, could still be utilized to enhance the possibilities to reliably fit genetic structure models. This will be a priority research area for us in the near future to benefit the whole research community interested in questions related to statistical learning of genetic population structure.

## Authors' contributions

All authors contributed equally in writing, analyses and method development.
